# Mixed News about the Bad News Game

**DOI:** 10.5334/joc.324

**Published:** 2023-10-09

**Authors:** Megan E. Graham, Brittany Skov, Zoë Gilson, Calvin Heise, Kaitlyn M. Fallow, Eric Y. Mah, D. Stephen Lindsay

**Affiliations:** 1 Psychology, Simon Fraser University, Burnaby, Canada; 2 Psychology, University of Victoria, Canada

**Keywords:** Fake news, social media, gameified inoculation, Bad News Game

## Abstract

Basol et al. (2020) tested the “the Bad News Game” (BNG), an app designed to improve ability to spot false claims on social media. Participants rated simulated Tweets, then played either the BNG or an unrelated game, then re-rated the Tweets. Playing the BNG lowered rated belief in false Tweets. Here, four teams of undergraduate psychology students each attempted an extended replication of Basol et al., using updated versions of the original Bad News game. The most important extension was that the replications included a larger number of true Tweets than the original study and planned analyses of responses to true Tweets. The four replications were loosely coordinated, with each team independently working out how to implement the agreed plan. Despite many departures from the Basol et al. method, all four teams replicated their key finding: Playing the BNG reduced belief in false Tweets. But playing the BNG also reduced belief in true Tweets to the same or almost the same extent. Exploratory signal detection theory analyses indicated that the BNG increased response bias but did not improve discrimination. This converges with findings reported by Modirrousta-Galian and Higham (2023).

## Mixed News about the Bad News Game

Wouldn’t it be nice if people who use social media were able to identify false posts as false? Toward that end, Roozenbeek and van der Linden (2019) developed the Bad News Game (BNG). The BNG is an online activity designed to “immunize” users against false posts on social media platforms such as Twitter. In analogy to inoculation, the BNG exposes participants to weakened examples of techniques used to disseminate misinformation, along with explanations of why those techniques are misleading, with the aim of building resistance to misinformation.

In a study of the Bad News Game, Basol et al. (2020) asked participants to rate the reliability of each of a set of simulated false and true news-related Tweets and then to play the BNG or an unrelated control game (a version of Tetris) before re-rating the same set of Tweets. They interpreted their findings as evidence that “Playing the BNG significantly improves people’s ability to spot misinformation techniques” (Basol et al., 2020, Abstract).

Here we report four parallel attempts to replicate (and extend) the Basol et al. (2020) study. The replications were conducted by teams of undergraduates at the University of Victoria in the spring of 2022. Basol et al. used a Qualtrics program (with a plug-in for the BNG), which they graciously shared with us. An important pedagogical aim of our project was to simulate cases in which different teams of scientists attempt to replicate a previously published study, so teams were allowed latitude in modifying the program (e.g., updating and “Canadianizing” the Tweets, clarifying the measure, etc.). For example, the Basol et al. (2020) participants rated the “reliability” of Tweets, whereas our teams instead asked for ratings of “truth” or “accuracy” (because those seemed simpler words for the psychological construct of central interest). Another difference was that Basol et al. collected participants’ ratings of confidence in each reliability rating, but we omitted the confidence ratings.

The most important difference between the original Basol et al. (2020) procedure and our replications related to true Tweets. The Basol et al. procedure included 3 true Tweets at pre-test and post-test, intermixed with the 18 false Tweets that were the focus of their interest. If the BNG improves ability to spot misinformation, then it should reduce belief in false Tweets more than it reduces belief in true Tweets. Using only 3 true Tweets would lead to low power to detect that interaction, but dramatically reducing the predominance of false Tweets might undermine the efficacy of the BNG inoculation. Balancing those two considerations, each of the four replications included 8 (rather than 3) true Tweets mixed among 24 (rather than 18) false Tweets. Unlike the Basol et al. study our planned analyses included ratings of both true and false Tweets. Thus, our studies were replications with extension (Brandt et al., 2014).

Our project addressed two main questions. Firstly, we aimed to assess the robustness of the Basol et al. (2020) finding that playing the BNG reduced ratings of the perceived reliability (truth) of false Tweets. Would that effect replicate across changes in time, source of participants, the Tweets, and the wording of the instructions and rating scales? Secondly, we sought to illuminate the mechanism of the BNG effect by determining whether the reduction in rated belief was specific to the false Tweets (as implied by the Basol et al. claim that the BNG “improves people’s ability to spot misinformation techniques”) or instead also comparably reduced belief in the true Tweets (which would indicate a bias effect rather than an improvement in sensitivity; cf. Modirrousta-Galian & Higham, 2023).

## Method

### Participants

Basol et al. (2020) analyzed data from 196 participants recruited from Prolific. Our participants were University of Victoria undergraduates who received optional bonus points in a psychology course. In the participant pool, about 75% identified as women and almost all were between 18 and 25 years old. It is not trivial to set the appropriate sample size for a replication (e.g., Simonsohn, 2015), especially given that we changed the design of the experiment by adding analyses of true Tweets. In any case, we were constrained by the schedule of the semester. Data collection continued until a pre-set deadline, with no pre-specified target *N*. Before exclusions, we attained *N* = 422, with approximately 105 participants in each group. After exclusions, each team had data from approximately 80 participants (see Table 1 for specifics), for a total of 353 participants across the four replications. Excluded participants were those who did not complete all questions in the study.

**Table 1:** Details of Each of the Four Replications.


TEAM	PARTICIPANTS	PARTICIPANTS (INOCULATION)	PARTICIPANTS(CONTROL)	SCALE	INDIVIDUAL DIFFERENCE MEASURE(S)

Bad News Bears	90	46	44	7-point	Hours/day on social media

Bikes	88	45	43	7-point	Social Desirability

Fake News Dudes	87	44	43	6-point	Social media use

pHackers	88	45	43	6-point	Hours/day on social media; Academic Year

**Total**	**353**	**180**	**173**		


### Materials

The BNG/Tetris portion of the procedure was identical to that used by Basol et al. (2020). Each team modified the informed consent and the instructions in the pretest and post-test portions of the experiment to update and Canadianize them. The four Qualtrics programs are available at https://osf.io/9v3kh/; below we review the most substantive changes.

1. Each team generated a set of 24 false and 8 true Tweets designed to closely mirror Tweets used by Basol et al. (2020) while making them current and suitable to the Canadian context.^1^ Some were modified versions of Tweets created by Basol et al., whereas others were newly created by student team members. These were pooled and each team selected from this pool 24 false and 8 true Tweets that they believed were best suited for the experiment. Each team’s Tweets slightly differed from others’ and can be found in their Qualtrics program.
2. Basol et al. (2020) measured perceived reliability as well as confidence in each reliability rating. As noted earlier, we instead collected ratings of truth or accuracy (although one team collected both a measure of truth and a measure of reliability). We dispensed with the confidence measure. Rozenbeek et al. (2022) reported that whether a participant was asked if a headline was “accurate” or “reliable” or “trustworthy” did not significantly affect their ratings.
3. Teams were instructed to modify the instructions and the end-point labels for the truth rating scale with the aim of validly measuring the intended psychological construct. The instructor had not intended students to change the number of scale points, but as shown in Table 2 some teams did so. The Basol et al. (2020) 7-point Likert scale is shown in Table 3.
4. Teams were told that they could collect one individual-difference measure after the post-test. For example, some teams chose to include a social desirability scale in their survey; others asked participants to rate their social media usage; one team collected two individual difference measures (see Table 1 and the Qualtrics programs). We have not explored relationships between these individual difference measures and the central measures of interest (but others are free to do so).

**Table 2:** Truth Rating Scales for True and False Tweets by Student Research Team.


RESEARCH TEAM	SCALE PROMPT	SCALE						

Bad News Bears	“How truthful do you find this post?”	Definitely untrue			Neutral/unsure			Definitely true

		1	2	3	4	5	6	7

Bikes	“How true or false is this post?”	Definitely false	Probably false	Maybe false	Neutral	Maybe true	Probably true	Definitely true

		1	2	3	4	5	6	7

pHackers	“How accurate do you find this post?”	Not at all		Neutral				Very

		1	2	3		4		5

Fake News Dudes	“How truthful do you find this tweet?”	Definitely false	Mostly false	Slightly false	Slightly true	Mostly true		Definitely true

		1	2	3	4	5		6

	“How reliable do you find this tweet?”	Definitely unreliable	Mostly unreliable	Slightly unreliable	Slightly reliable	Mostly unreliable		Definitely reliable

		1	2	3	4	5		6


**Table 3:** Basol et al.’s Reliability and Confidence Rating Scales for True and False Tweets.


BASOL ET AL.	SCALE PROMPT	SCALE						

	“How reliable do you find this post?”	Not at all			Neutral			Very

		1	2	3	4	5	6	7

	“How confident are you in your judgement?”	Not at all			Neutral			Very

		1	2	3	4	5	6	7


### Procedure

The study was approved by the University of Victoria Human Research Ethics Board. A posting on the University of Victoria SONA system invited participants to complete the online experiment, starting on March 24, 2022. A script rotated assignment of volunteers to the four replications. Before beginning the study, participants were presented with an informed consent page. If the individual consented to participate (by clicking a check-box indicating that they agreed to participate in the study as described), they were taken to the instructions for the initial ratings of Tweets. Participants were randomly assigned to either the Inoculation condition (BNG) or the Control condition (Tetris). We did not conduct attention checks. Data collection stopped on April 7, 2022.

## Results

**Figure 1:**
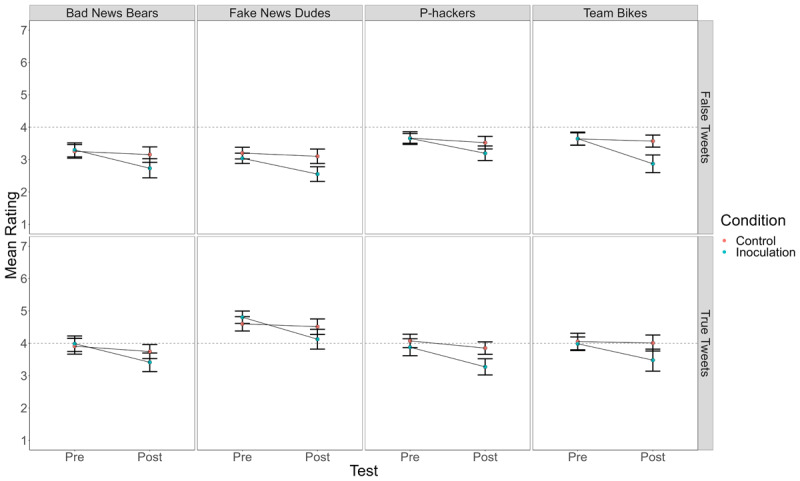
Mean Truth Ratings for True and False Tweets Before and After Playing the Bad News Game (Inoculation) or Tetris (Control) in Each of the Four Replication Attempts.

**Figure 2:**
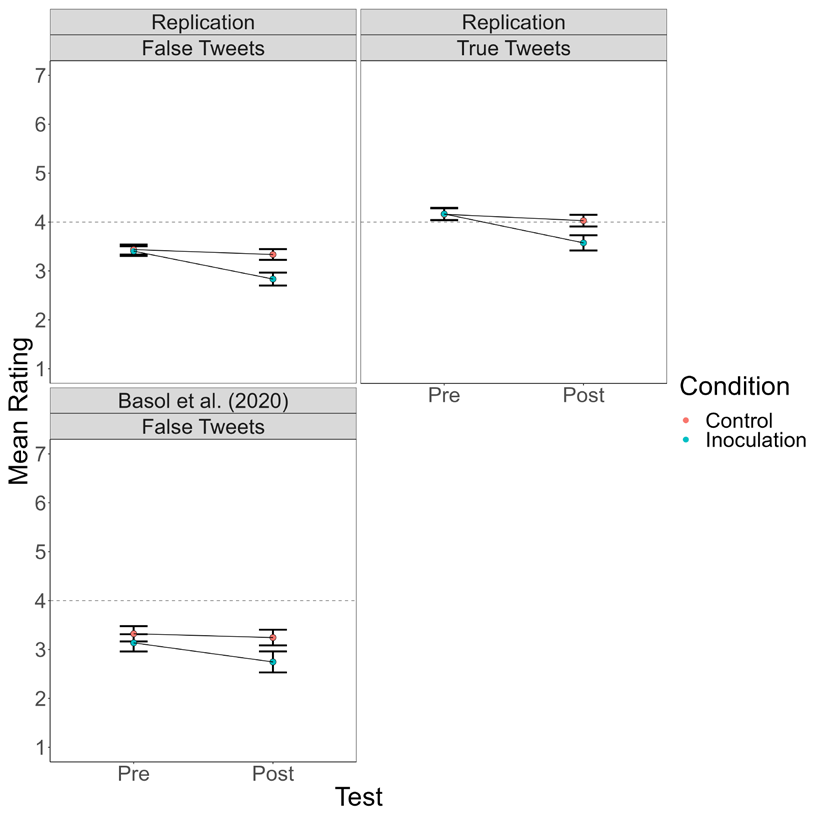
Mean Truth Ratings for True and False Tweets Before and After Playing the Bad News Game (Inoculation) or Tetris (Control) Across the Four Replications and for False Tweets in the Basol et al. (2020) Data.

Because some teams used rating scales with different numbers of points (see Table 2), we first rescaled all ratings to a 7-point scale. Specifically, for the 1 to 5 scale, 1 remained 1, 2 became 2.5, 3 became 4, 4 became 5.5, and 5 became 7; for the 1 to 6 scale, 1 remained 1, 2 became 2.2, 3 became 3.4, 4 became 4.6, 5 became 5.8, and 6 became 7. For each team, we analyzed the ratings in a 2 (BNG vs. Tetris) × 2 (pre-test vs. post-test) × 2 (true vs. false tweet) mixed-model analysis of variance (ANOVA) using JASP 0.16.1. The results of those analyses are in Table 4. Figure 1 presents a line graph of mean ratings by condition for each team’s replication. Figure 2 shows ratings by condition in the data combined across teams, along with the data from Basol et al. (2020) for comparison.

**Table 4:** ANOVA Results Tables for Each Team.


TEAM	EFFECT	*F*	*p*	*n* ^2^ *p*

Bad News Bears	True/False	93.22	<.001	.514

(df 1, 88 for all)	True/False × Condition	<1.00	.647	.002

	Pre/Post	38.74	<.001	.306

	Pre/Post × Condition	15.02	<.001	.146

	True/False × Pre/Post	<1.00	.529	.005

	True/False × Pre/Post × Condition	<1.00	.605	.003

	Condition	1.09	.300	.012

Fake News Dudes	True/False	379.31	<.001	.817

(df 1, 85 for all)	True/False × Condition	2.82	.097	.032

	Pre/Post	46.41	<.001	.353

	Pre/Post × Condition	24.38	<.001	.223

	True/False × Pre/Post	2.19	.143	.025

	True/False × Pre/Post × Condition	2.79	.098	.032

	Condition	3.09	.083	.035

pHackers	True/False	15.28	<.001	.151

(df 1, 86 for all)	True/False × Condition	1.58	.212	.018

	Pre/Post	34.97	<.001	.289

	Pre/Post × Condition	8.53	.004	.090

	True/False × Pre/Post	3.48	.065	.039

	True/False × Pre/Post × Condition	<1.00	.513	.005

	Condition	6.42	.013	.069

Team Bikes	True/False	33.07	<.001	.278

(df 1, 86 for all)	True/False × Condition	<1.00	.657	.002

	Pre/Post	28.37	<.001	.248

	Pre/Post × Condition	19.10	<.001	.182

	True/False × Pre/Post	5.22	.025	.057

	True/False × Pre/Post × Condition	4.33	.040	.048

Condition		8.04	.006	.086


The ratings for false Tweets replicated the findings of Basol et al. (2020). That is, in each team, rated-truth of false Tweets significantly dropped from pre-test to post-test for subjects who played the BNG, but not for those who played Tetris. A new finding is that truth ratings for true Tweets followed a similar pattern, significantly dropping among subjects who played the BNG but not for those who played Tetris. See Table 6.

**Table 6:** Effect Size Estimates (Cohen’s *d*) for the Difference Between Pre-test and Post-test Ratings (and the Associated 95% Confidence Interval) for True and False Tweets as a Function of Treatment Group.


TEAM	VALUE OF COHEN’S *d*	

	FALSE TWEETS	TRUE TWEETS

Bad News Bears	0.55 (CI 0.23)	0.58 (CI 0.22)

Fake News Dudes	0.62 (CI 0.23)	0.56 (CI 0.23)

pHackers	0.52 (CI 0.22)	0.56 (CI 0.23)

Team Bike	0.64 (CI 0.23)	0.36 (CI 0.22)

All UVic Teams	0.58 (CI 0.12)	0.51 (CI 0.11)


As an index of effect size, Basol et al. (2020) compared the reduction from pre- to post-test ratings of false Tweets among subjects who played the BNG versus those who played Tetris. They reported a Cohen’s *d* = 0.60. As shown in Table 7, in our replications the estimated size of that same effect ranged from 0.56 to 1.23. So, the key Basol et al. finding replicated strongly and consistently.

**Table 7:** Change in Truth Ratings on False Tweets from Pretest to Post-test as Function of Training Condition (BNG vs. Tetris).


TEAM	*t*	*df*	*p*	COHEN’S *d*	95% CI FOR COHEN’S *d*	

					LOWER	UPPER

Bad News Bears	3.88*	62.59	<.001	0.81	1.25	0.37

Fake News Dudes	4.16	85.00	<.001	0.89	1.33	0.45

pHackers	2.64*	65.47	.01	0.56	0.99	0.13

Team Bikes	5.79*	72.96	<.001	1.23	1.69	0.76


*Welch test reported due to heterogeneity of variance.

If playing the BNG improved ability to spot misinformation, then playing the game would reduce truth ratings of false Tweets more than it reduced truth ratings of true Tweets. That would give rise to BNG/Tetris × Pre/Post × Truth/False interaction. In Team Bikes, that three-way interaction was (barely) significant, *F*(1, 86) = 4.33, *p* = .04, indicating that the effect of playing the BNG tended to be greater for false Tweets than for true Tweets. There was also a small and non-significant difference in that direction for the Fake News Dudes team, *F*(1, 85) = 2.79, *p* = .10. In the remaining two replications, there was no evidence of a greater effect of playing the BNG on ratings of false than of true Tweets (*F* < 1).

We also conducted an omnibus ANOVA that added team as a between-subjects variable. The results of that ANOVA are shown in Table 4. Note the tantalizing non-significant *p* value for the 4-way interaction, *p* = .054. That suggests that there may have been variations across the replications in the extent to which playing the BNG differentially affected ratings of true versus false Tweets. But in that omnibus ANOVA the crucial three-way interaction (which would indicate a greater effect of the treatment on ratings of false than true Tweets across experiments) was *F* < 1.

## ROC analysis

**Figure 3:**
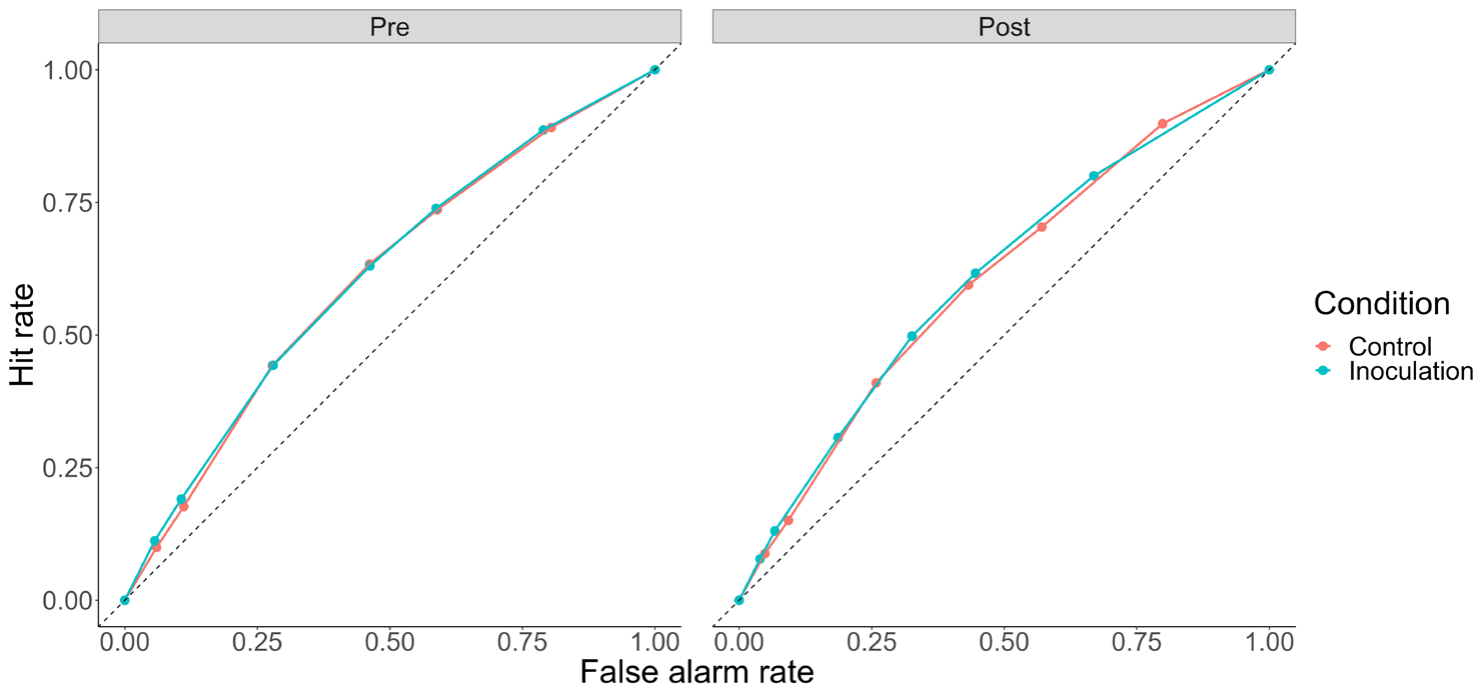
ROC Curves by Condition and Pre/Post. *Note*: Dashed line represents chance sensitivity, with curves bowing further from the dashed line representing higher sensitivity.

In a recent meta-analysis of gamified inoculation interventions, Modirrousta-Galian and Higham (2023) used *receiver operating characteristic* (ROC) analyses to assess participants’ ability to discriminate true and false tweets. ROC analysis is based on signal detection theory (Paterson et al., 1954). It assumes that participants evaluate the subjective strength of evidence for (in this case) truth of true and false tweets against an internal criterion, and make a judgment of truthfulness if the subjective evidence exceeds the criterion. Following Modirrousta-Galian and Higham, we used participants’ truth ratings as indices of a range of internal criterion values and computed the rate of hits (“true” responses to true tweets) and false alarms (“true” responses to false tweets) when each point on the scale is treated as the cutoff for truth.^2^ Then we constructed ROC curves for the series of hit and false alarm rates and calculated area under the curve (AUC) as a response-bias-free measure of sensitivity or ability to discriminate between true and false Tweets. We constructed four ROC curves, one for each of combinations of BNG/Tetris and Pre/Post. These ROC curves are shown in Figure 3.

Although the lack of differences between conditions is visually apparent, we also conducted formal analyses of AUC. Like Modirrousta-Galian and Higham (2023), we computed AUC as:


\[
{\boldsymbol{AUC = 0.5\mathop \sum \limits_{k = 0}^n (H{R_{k + 1}} + H{R_k})(FA{R_{k + 1}} + FA{R_k})}}
\]

Where *k* denotes each of the *n* (seven) criteria plotted on the curve, HR refers to the hit rate at that criterion, and FAR refers to the false alarm rate at that criterion. There was no significant effect of inoculation condition on AUC, *F*(1, 345) = .007, *p* = .93, no significant effect of pre/post on AUC, *F*(1, 345) = .14, *p* = .70, and no significant interaction, *F*(1, 345) = .03, *p* = .85. To assess evidence *against* an effect of the BNG on sensitivity, we conducted a Bayesian *t* test comparing post-manipulation AUC in the control and inoculation conditions. The corresponding Bayes Factor against a difference was 8.43, indicating that no effect of the BNG was around eight times more likely than the alternative (“moderate” evidence according to Lee & Wagenmakers, 2013). These results – evidence against a sensitivity increase with the BNG – agree with the conclusions of Modirrousta-Galian and Higham (2023).

## Response bias

Both our data and the recent meta-analysis by Modirrousta-Galian and Higham (2023) suggest that the BNG affected response bias but had little if any effect on sensitivity. Specifically, it may be that the BNG simply increased response bias, making participants less likely to judge Tweets as true regardless of their veracity. Like Modirrousta-Galian and Higham (2023), we conducted exploratory analyses of response bias, operationalized as *B”D*, which is computed using the hit (HR) and false alarm rates (FAR) at each scale point via the following formula (Donaldson, 1992; Modirrousta-Galian & Higham, 2023):


\[
{\boldsymbol{B^{\prime\prime}D = \frac{{(1 - HR) (1 - FAR) - (HR)(FAR)}}{{(1 - HR) (1 - FAR) + (HR)(FAR)}}}}
\]

**Figure 4:**
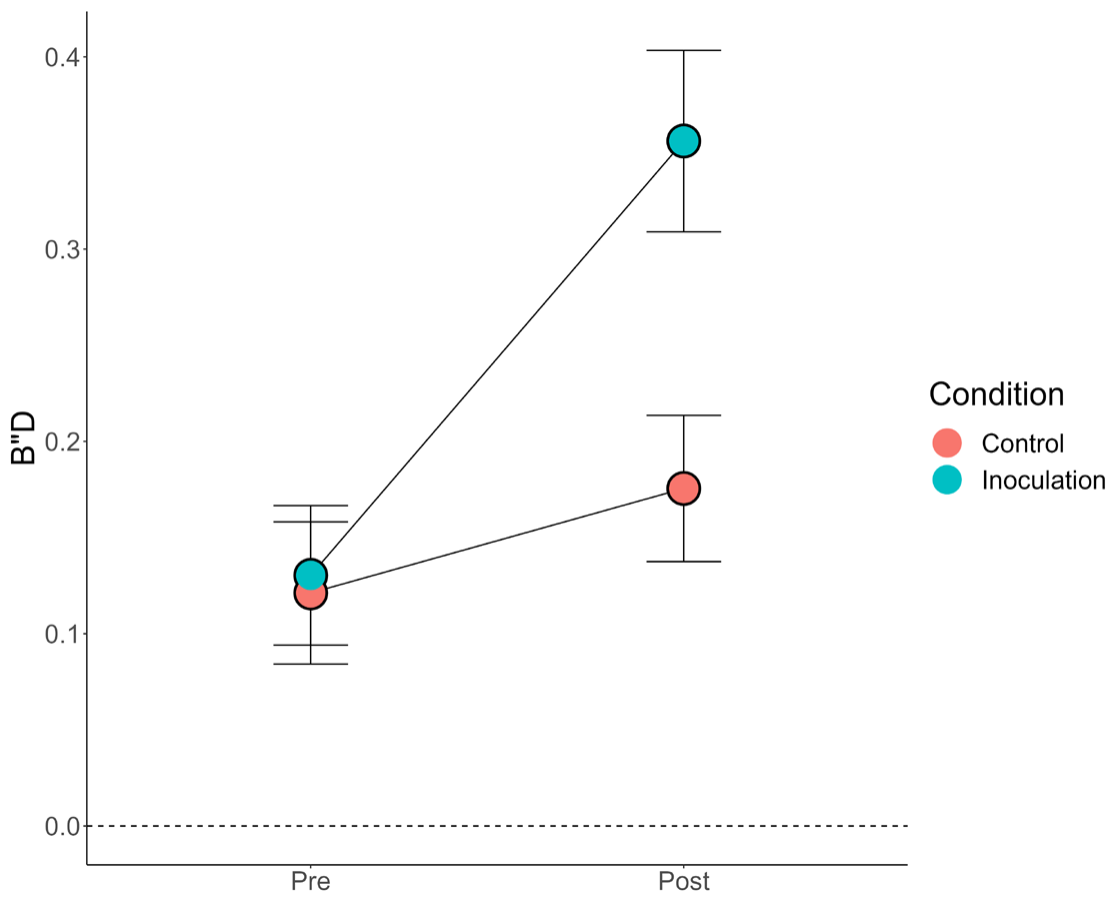
*Response Bias (B”D) by Condition and Pre/Post*. *Note*: Error bars = 95% CIs (between-subjects). Dashed line at 0 *B”D* indicates “neutral” response bias.

*B”D* provides an accurate measure of response bias that can be applied to collapsed/grouped data across the full range of sensitivity performance (see Modirrousta-Galian & Higham, 2023 for details of this metric). Based on the differences in mean ratings, we might expect a higher post-manipulation *B”D* in the inoculation condition. That is indeed what we found, as shown in Figure 4.

There was a significant main effect of both condition, *F*(1, 345) = 13.13, *p* < .001, *η^2^_g_* = .03,^3^ and pre/post, *F*(1, 345) = 138.74, *p* < .001, *η^2^_g_* = .06, qualified by an interaction between the two, *F*(1, 345) = 51.46, *p* < .001, *η^2^_g_* = .03. The BNG increased response bias relative to the Tetris control. Interestingly, pre-manipulation, participants tended to be conservative (a general distrust of the presented Tweets, possibility justified given the higher number of false Tweets). Even participants in the control condition tended to be slightly more conservative when rating the Tweets for a second time, t(341.73) = 2.00, p = .045, d = .22. But, the critical finding was in accordance with our analyses of mean ratings and the similar analyses conducted by Modirrousta-Galian and Higham (2023): The BNG made participants significantly more conservative but not significantly more accurate.^4^

## Discussion

It is not possible to conduct an exact replication of a previously conducted experiment. At minimum, the original and replication are conducted at different times. Our studies differed from the Basol et al. (2020) experiment in *many* ways. Basol et al. collected their data at least 2 years earlier, in a pre-COVID world. They sampled from Prolific, whereas we sampled University of Victoria psychology undergraduates. We made major changes to the materials, procedure, and design (e.g., updated and Canadianized the Tweets, increased the number of Tweets and especially the number of true Tweets, measured perceived “truth” rather than “reliability,” and added analyses of ratings of true Tweets to the design). Each team was given latitude as to how to implement the agreed-upon changes, so no two replications were the same. It is not straightforward to specify which aspects of a procedure must be reproduced with high fidelity to have a high likelihood of replicating the original findings (Buzbas et al., 2022). Each team sought to conduct a good-faith extended replication that was true to the conceptual aims of the Basol et al. (2020) study, but they did so as undergraduate students operating under tight time constraints.

Despite the myriad ways our studies differed from the original, our results for false Tweets mirrored those of Basol et al. (2020). In each replication, playing the Bad News Game substantially and significantly reduced ratings of false Tweets (see Table 7 for effect size estimates). This indicates considerable robustness of the effect Basol et al. reported.

The less good news is that the Bad News Game also reduced ratings of the truth of true Tweets. That effect, too, was substantial and significant in each replication study. There were hints that the BNG had a slightly bigger impact on the perceived truth of false than true Tweets in two of the studies. But that 3-way interaction was statistically significant in only one of the studies, and mean-level and ROC analyses across all four experiments provided no support for the hypothesis that playing the BNG improved discrimination between true and false Tweets across these studies. Instead, the BNG resulted in significantly more conservative responding, with little evidence for an increase in discrimination – even in the sample in which the mean-level analysis was suggestive of an interaction.

Participants’ truth ratings varied across our four teams. As shown in Table 5, there was a significant main effect of team on truth ratings. Moreover, in two teams the BNG was directionally associated with a bigger pre-post reduction in truth ratings for false than for true Tweets (although the *p* value for that 3-way interaction was .04 in one case and .10 in the other). Differences in truth ratings between teams might just be chance – indeed, in the omnibus analysis with team as a factor, the four-way interaction was not (quite) statistically significant. But it may be that differences between teams in the Tweets and/or rating scales and/or instructions affected truth ratings and perhaps even modulated the effect of the BNG. Allowing variability across teams in how the replication was implemented was not a bug but rather a feature of the project and it is not surprising that it raises questions for future study.

**Table 5:** Results of a Mixed Model Omnibus Analysis of Variance of Ratings, with True/False Tweets and Pre/Post Ratings as Repeated Measures and Condition and Team as Between-Subjects Factors.


WITHIN SUBJECTS EFFECTS			

EFFECT	DF	F	P

TrueFalse	1	388.55	<.001

TrueFalse ✻ Condition	1	0.53	.467

TrueFalse ✻ team	3	56.75	<.001

TrueFalse ✻ Condition ✻ Team	3	1.50	.214

Residuals	345		

PrePost	1	142.59	<.001

PrePost ✻ Condition	1	63.03	<.001

PrePost ✻ Team	3	0.03	.994

PrePost ✻ Condition ✻ Team	3	0.70	.554

Residuals	345		

TrueFalse ✻ PrePost	1	0.81	.368

TrueFalse ✻ PrePost ✻ Condition	1	0.01	.928

TrueFalse ✻ PrePost ✻ Team	3	3.68	<.05

TrueFalse ✻ PrePost ✻ Condition ✻ Team	3	2.57	.054

Residuals	345		

**BETWEEN SUBJECTS EFFECTS**			

**EFFECTS**	**DF**	**F**	**P**

Condition	d	15.67	<.001

Team	3	3.76	.011

Condition ✻ Team	3	0.50	.681

Residuals	345		


A post-hoc sensitivity analysis using summary statistics (means, SDs, correlations between within-subjects conditions) from our data combined across the four studies revealed that we had limited power to detect subtle BNG effects. The primary effect of interest was the three-way interaction between condition (BNG/Tetris), Tweet veracity (true/false), and time (pre-manipulation/post-manipulation), which would be significant if the BNG reduced ratings of false Tweets more than ratings of true Tweets, relative to Tetris. For example, suppose that in this population with this procedure the BNG reduces belief in false Tweets by an average of .57 on the 7-point scale (about what we observed) but does not affect belief in true Tweets. Our sensitivity analysis^5^ revealed that we only had power of about .5 to detect that interactive pattern. Of course, power would be even lower to detect a weaker interaction (e.g., one in which playing the BNG reduced belief in true Tweets half as much as it reduced belief in false Tweets, see Supplementary Material 1). Our claim is not that the BNG reduces belief in true and false Tweets to exactly the same degree. But the difference in post-manipulation ratings of true Tweets across conditions (Figure 2, panel 2) and the difference in pre-post BNG ratings of true Tweets provide compelling evidence that the BNG reduced belief in the true Tweets. Perhaps this reduction was slightly smaller than that for the false Tweets.

Modirrousta-Galian and Higham (2023) re-analyzed data from five experiments that used “inoculation” interventions, including the data from Basol et al. (2020). Their new analyses used signal detection measures of sensitivity (i.e., ability to discriminate true and false Tweets) and response bias (i.e., general tendency toward judging Tweets as true or as false). For the re-analysis of Basol et al. (2020), Modirrousta-Galian and Higham included the data from the three true Tweets that Basol et al. had included in their procedure but not in their report. The signal detection analysis indicated that the BNG affected response bias not sensitivity in the Basol et al. study and in other four data sets published as evidence of “inoculation” against misinformation. We followed their lead and conducted analogous ROC analyses of our data, which yielded converging evidence that playing the BNG led to conservative response bias but had little if any effect on sensitivity.

It is unclear to what extent the BNG affected participants’ subjective experiences of the Tweets (e.g., decreased perceptions of truth) versus merely affected their use of the rating scales (i.e., scale recalibration). That is, it could be that the BNG shifted response criterion upward and/or that it shifted perceived evidence of truth downward (Modirrousta-Galian & Higham, 2023). Future studies might probe these possibilities with qualitative post-judgement questions or multiple judgment scales.

A potential limitation of our study is that our Tweets may not be representative of real-world true and false Tweets. In particular, it might be that our true Tweets had more of the features of false Tweets that are targeted by the BNG than do most real-world true Tweets. We did not set out to create true Tweets that masqueraded as false Tweets, but we did not conduct a content analysis to assess that issue. The representativeness of the false Tweets used by Basol et al. may also be open to question. Baseline Tweet plausibility is important to consider – obviously true items and obviously false items are unlikely to be affected by inoculation manipulations (Modirrousta-Galian & Higham, 2023). Alternatively, a combination of too-false false items and too-ambiguous true items could result in an apparent negative effect of the BNG (reduction in ratings for true but not false items).

We explored the distributions of the initial truth ratings of our Tweets (collapsed across BNG and control conditions). The proportion of Tweets rated on average as “initially false” (≤ 3) was significantly higher for false than true Tweets (.35 vs. .13, *p* = .006) and the proportion of Tweets rated as “initially true” (≥ 5) was significantly higher for true than false Tweets (.19 vs. .04, p = .001). But most true and false Tweets fell in the “ambiguous” range (rating = 4): .61 for false Tweets, .67 for true Tweets, p = .53) (see Supplementary Material 3 for details).

**Figure 5:**
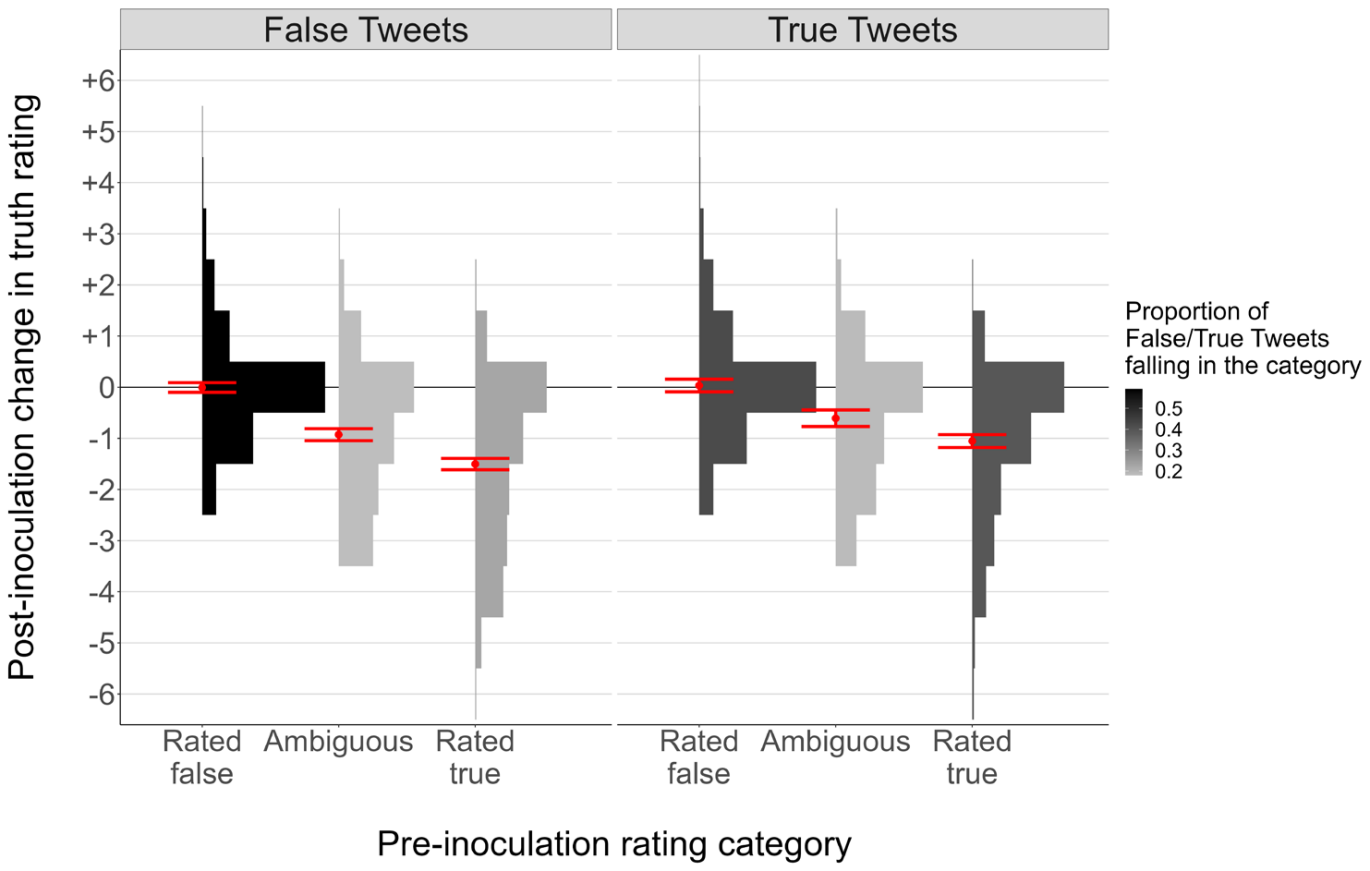
Change in Truth Ratings as a Function of Tweet Ground Truth and Initial Truth Ratings. *Note*: Red points = model-predicted means, red error bars = model-predicted 95% CIs, histograms = relative frequency of each post-inoculation effect in each pre-inoculation rating category.

The effects of the BNG might differ as a function of initial truth ratings. For example, it may be that the BNG primarily reduces trust in Tweets of ambiguous veracity (that participants aren’t sure about), whereas Tweets initially viewed as true or false are unaffected. Because most of our true and false Tweets fell in the “ambiguous” range, it is possible that we did not have enough “high-perceived-truth” Tweets to see that the BNG has no effect on judgments of these Tweets. To test the possibility of ambiguity-based effects on the BNG’s efficacy, we examined inoculation-based reductions in ratings as a function of the ambiguity cutoffs described previously. If the BNG affects mainly ambiguous Tweets, we should see smaller post-inoculation reductions for “initially rated as true” Tweets (≥ 5) and “initially rated as false” (≤ 3) Tweets than for Tweets initially rated 4. This was not the case: A mixed-effects linear model of inoculation effects (at the item-level, including random intercepts by participant) resulted in significant main effects of ground truth, *χ^2^*(1) = 44.07, *p* < .001 and p*re-inoculation rating category*, χ2(2) = 1411.23, p < .001, and an interaction between the two, χ2(2) = 27.36, p < .001. Crucially, and as is evident from Figure 5, the post-inoculation reduction in *truth* ratings was significantly larger for items initially rated as true than for items initially rated as *a*mbiguous, both for true Tweets, z = 5*.2*0, p < .0*0*1, and false Tweets, z = 10.08, p < .001. In a subsequent analysis in which we restricted “initially rated as true” to ≥ 6 it was still the case that the reduction in truth ratings associated with playing the BNG was greater for those items than for items initially rated as ambiguous (4), and this held for both *t*rue items and false items (both ps < .001). Thus, the BNG reduced belief in the truth of true items that subjects initially indicated they were at least pretty sure were true.

These results suggest that the BNG made participants more skeptical of Tweets they initially viewed as more likely to be true than Tweets they were unsure about. Interestingly, items participants initially thought likely to be false were the least affected by the BNG. These results reinforce the idea that the BNG (at least in its original form) promotes a general conservative response bias, with the “skeptical shift” larger for information participant**s** initially placed more stock in. Of course, it is also possible that the increased reduction for “initially rated as true” Tweets was merely because Tweets with higher ratings had more room for downward shifts. But it is clear that the BNG reduced truth ratings for true Tweets that participants initially rightly judged to be true.

Because this result is largely independent of the fact that we had more false than true Tweets with low perceived truth ratings (35% vs. 13%), we think it unlikely that this specific configuration of true/false/ambiguous Tweets explains our results. But the potential sensitivity of inoculation manipulations to baseline believability revealed in these analyses suggests that interpretations of inoculation findings must be nuanced and context-sensitive. Based on these results, if one had a Tweet set with false Tweets initially perceived as true and true Tweets initially perceived as ambiguous or false, one might observe an apparent beneficial effect of the BNG purely as a result of response bias. Or, given the same underlying response bias, if the majority of both true and false Tweets were initially perceived as true, one might observe no effect of the BNG, etc. Item-level and signal detection analyses, like those performed by ourselves and Modirrousta-Galian and Higham (2023) are essential for a complete picture of these manipulations.

Relatedly, there is some evidence that the ratio of true/false items matters in inoculation experiments. For instance, Maertens et al. (2021) found evidence that the BNG increased reliability ratings of true items when there were equal numbers of true and false items but not when most items were false. It is possible that in majority-false stimulus sets (like our experiments and the Basol et al., 2020 experiments), participants adopt a generally conservative response tendency (Maertens et al., 2021). Modirrousta-Galian and Higham (2023) suggested that when false Tweets substantially outnumber true Tweets, participants may gradually pick up on that fact, which might in turn encourage adoption of a conservative response criterion. This is consistent with our observation of more conservative responding on the post-test than on the pre-test in both the BNG and Tetris conditions. The more pronounced shift for the BNG condition further suggests that the manipulation might amplify or reinforce this general sense. Of course, it may also (or instead) be important to ensure that the number of items per condition is sufficient to yield adequate statistical power. And if an unbalanced true/false ratio leads to general response tendencies, it is possible that these tendencies may be exacerbated with larger stimulus sets (such as our 32-Tweet sets).

That playing the BNG can have different effects—reductions, no change, or increases in belief ratings—with different designs and stimulus sets suggests that inoculation manipulations may increase discriminability under some conditions. Indeed, Iyengar, Gupta, and Priya (2023) reported that the BNG substantially improved discrimination (although Modirrousta-Galian & Higham (2023) suggested that a failure to counterbalance pre-test and post-test items casts some doubt on this result). But on balance, the evidence seems to suggest that the original BNG affects response bias but not discrimination. If there are conditions under which interventions such as the BNG improve discriminability, it is not clear what those conditions are, nor how well they map onto the conditions that social media consumers typically encounter (e.g., what are the typical true/false ratios in the average Twitter feed, how “true” are average true Tweets, how “false” are average false Tweets?).

In the absence of strong theoretical grounding and a priori predictions about specific effects of particular design choices, we urge caution in making claims about the broad generalizability of our results (and those of other inoculation studies). Our results mirror other findings that indicate response bias as an explanation for the pattern of responding seen after playing the BNG, but future experiments manipulating these methodological factors in theoretically informed ways could further elucidate the conditions under which inoculation manipulations are likely to improve discrimination. For example, Roozenbeek (Personal communication, 25 August 2023) described research by Leder et al. (2023) in which the BNG is followed by a feedback exercise designed to discourage participants from being overly skeptical. Reportedly, that procedure enhanced discrimination.

Even if a gamified inoculation procedure is shown to enhance discrimination, we would urge caution in generalizing the findings to practical real-world settings. In those experiments, subjects rate Tweets, get training or a filler activity, and then re-rate the initial Tweets. The whole process typically unfolds over a matter of minutes and in the context of overt demand characteristics for participants in the Inoculation condition. Would participants who played the BNG be more skeptical of posts they encountered on × the next day? Maybe, but we doubt it. We suspect that achieving that sort of generalizability would require much more extensive training aimed at helping participants develop skills for seeking additional probative information, as advocated by Brodsky et al. (2021) and Caulfield (2019).

## Data Accessibility Statement

The materials (including informed consent), data, and analysis scripts for the studies reported here are available on the Open Science Framework, https://osf.io/9v3kh/.

## Additional File

The additional file for this article can be found as follows:

10.5334/joc.324.s1 Supplementary File. Supplementary Materials 1 to 3.

## Footnotes

The Tweets for each group are on OSF: Bad News Bears, https://osf.io/v5mpx; Fake News Dudes, https://osf.io/uzn8s; P-Hackers, https://osf.io/kf7vu; Bikes, https://osf.io/pnw2a.

For these analyses, we used the 1–7 scale and rescaled ratings for the group datasets that used fewer scale points.

Effect size measure is generalized eta squared.

Because we found the strongest support for inoculation effects in the Team Bikes dataset, we conducted the ROC/AUC and B”D analyses in this dataset. Results were similar – evidence against an increase in sensitivity and evidence for an increase in response bias with the BNG (although reduced power to detect effects in this restricted dataset should be taken into account). These analyses can be found in Supplementary Material 2.

Conducted using the Superpower R package (Lakens & Caldwell, 2021).
