# Supplementary material for: Mixed News about the Bad News Game
Source: J Cogn. 2023 Oct 9;6(1):58. doi: 10.5334/joc.324 (PMC10573624; doi:10.5334/joc.324)
Supplement: Supplementary File. — Supplementary Materials 1 to 3. [file joc-6-1-324-s1.pdf]

Supplementary Material

1. **Sensitivity Analysis**
   a. **Sensitivity curve**

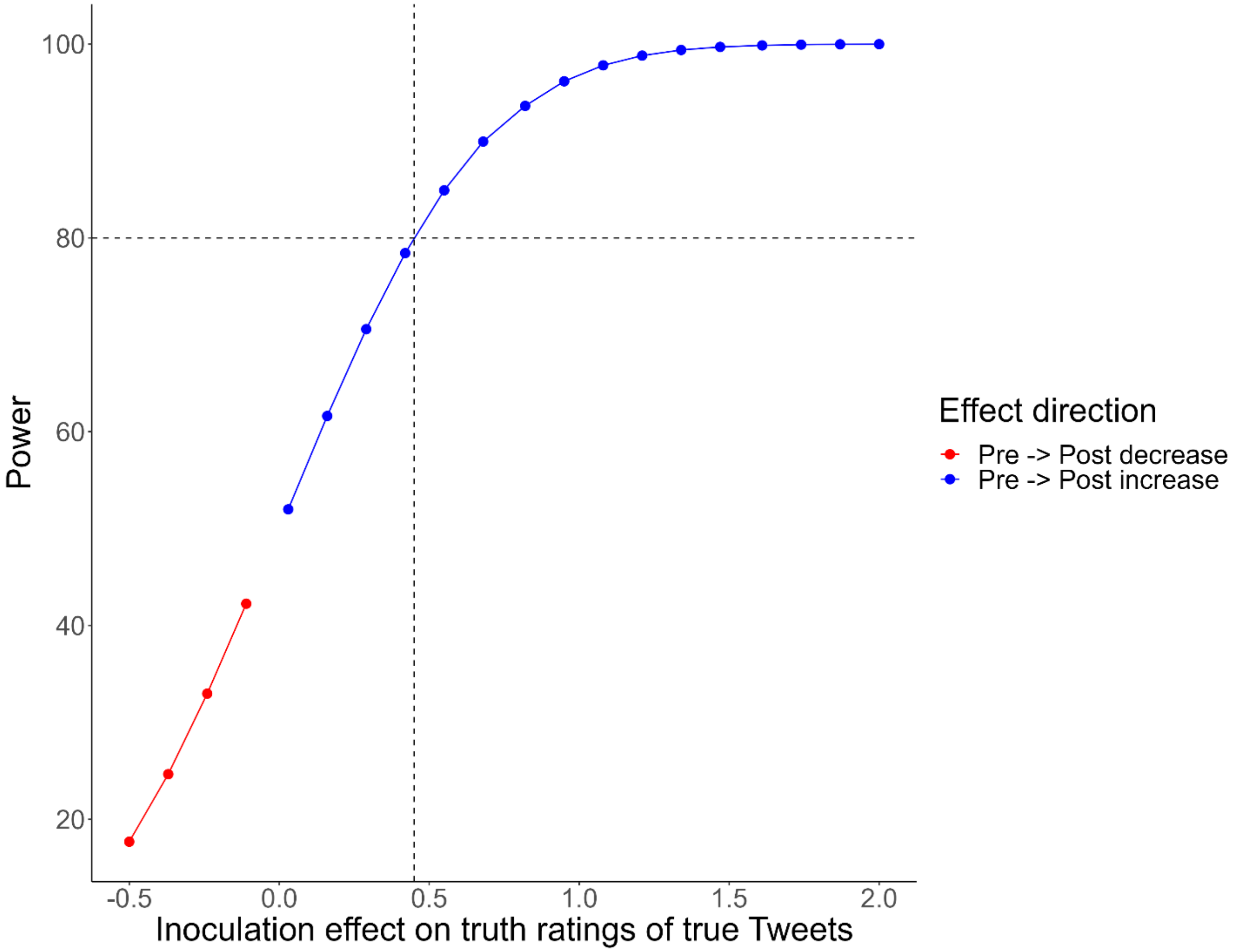

Note. Effect size metric is in units of the *Pre -> Post decrease in truth ratings for false Tweets in the Inoculation/BNG condition*. E.g., -0.5 implies that the *Pre -> Post decrease in truth ratings for true Tweets in the Inoculation/BNG condition* is ½ that of the decrease for false Tweets, and 2.0 implies that the *Pre -> Post increase in truth ratings for true Tweets in the Inoculation/BNG condition* is twice the magnitude of the decrease in truth ratings for false tweets.

b. Hypothetical cell means and SDs

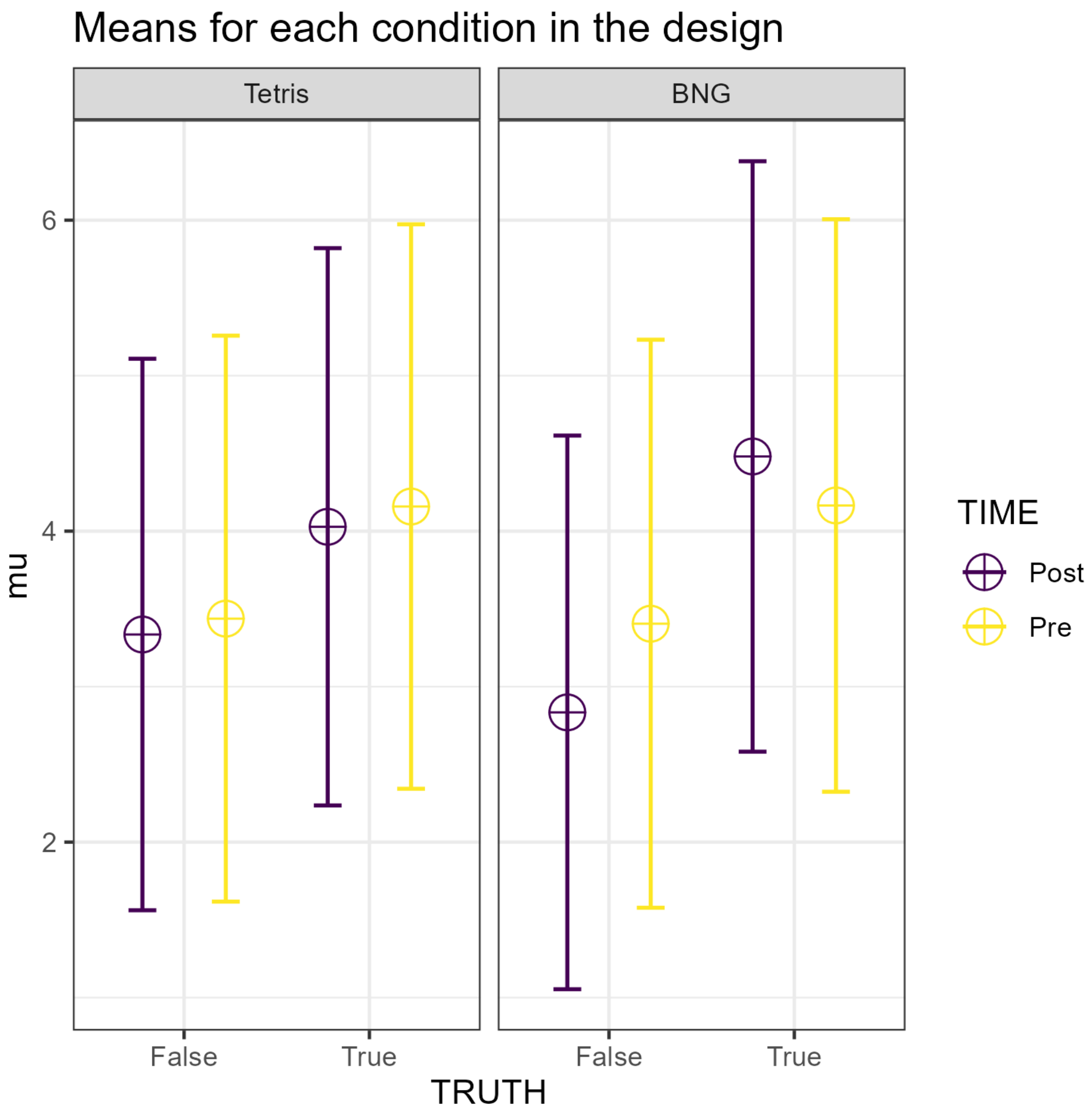

c. Hypothetical cell means and SDs for the interaction described in-text

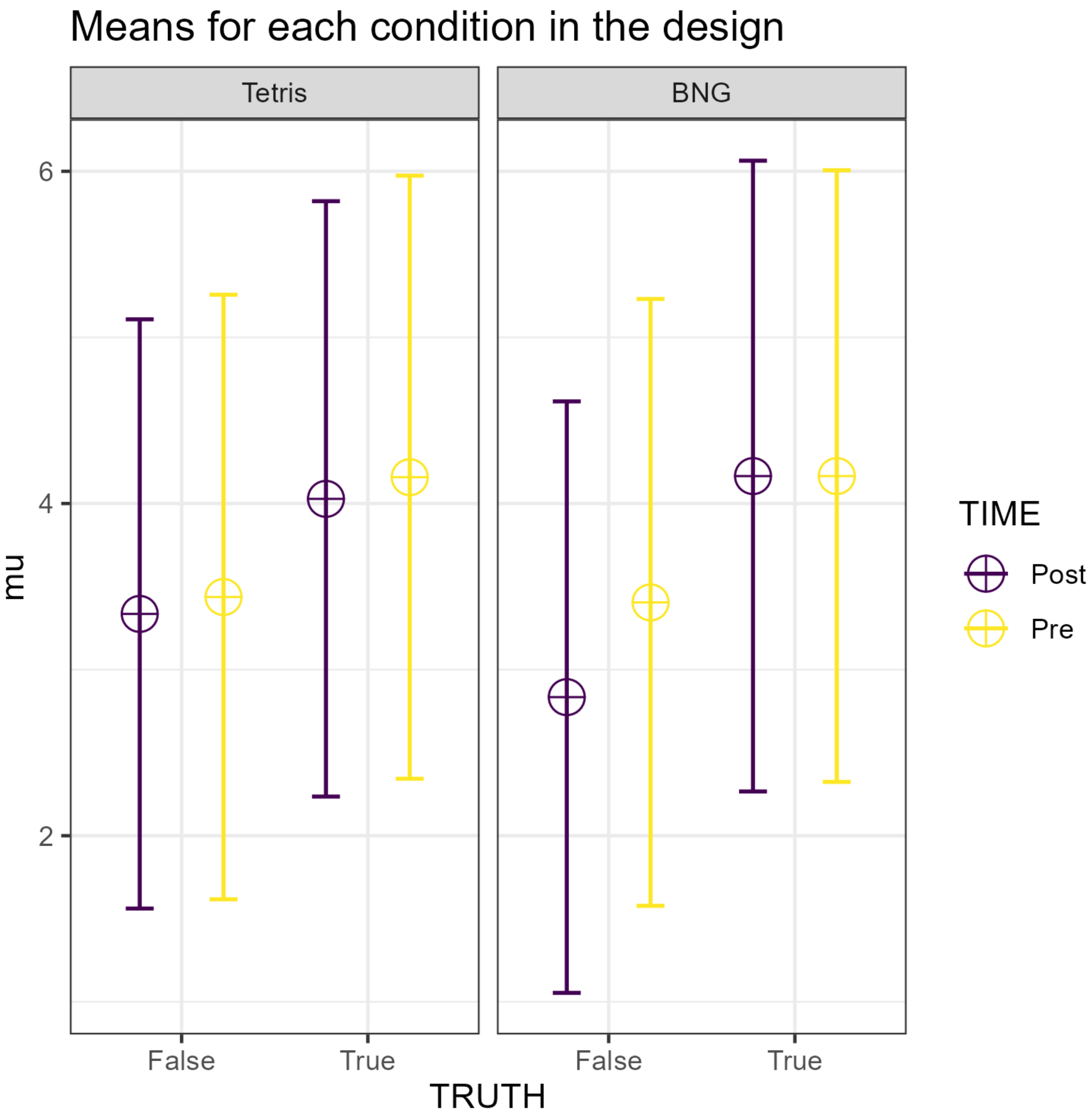

2. **SDT analyses of “Team Bikes” data**
   a. **ROC/AUC analysis**

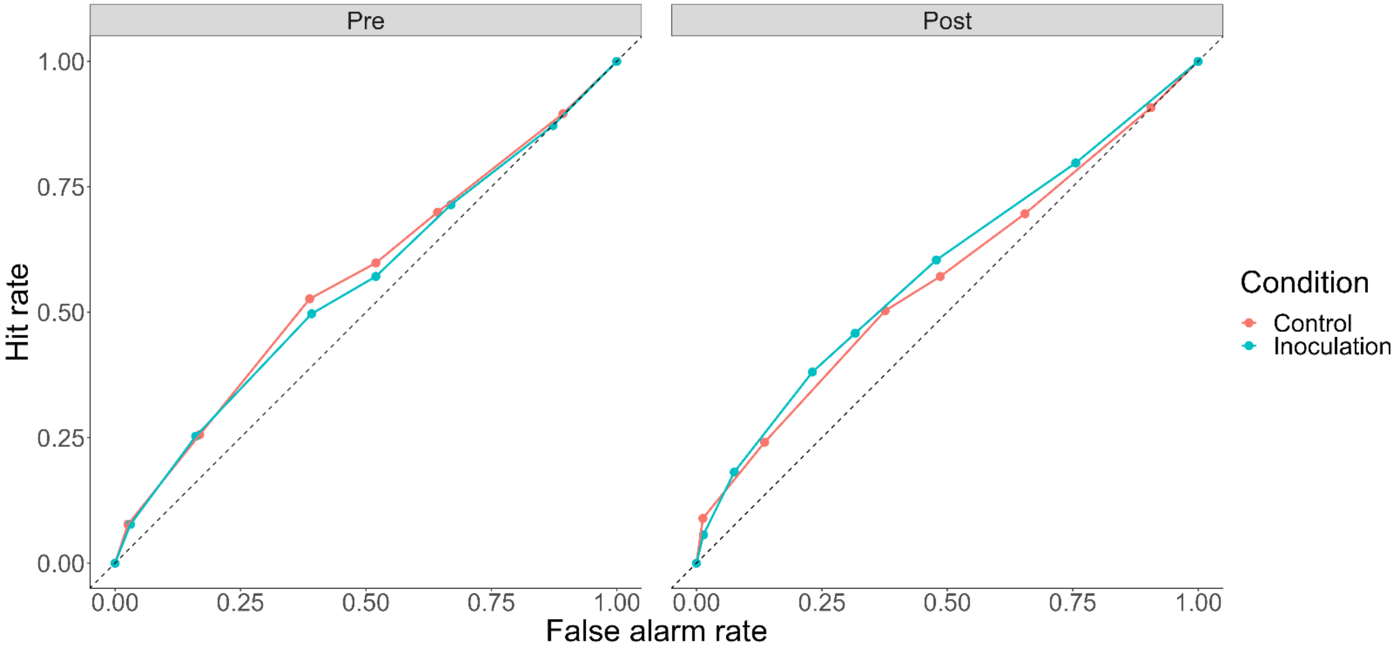

Non-sig. effect of condition: $F(1, 82) = .03$, $p = .87$

Sig. effect of pre/post: $F(1, 82) = 6.61$, $p = .01$

Non-sig. interaction: $F(1, 82) = 2.40$, $p = .13$

Evidence against effect of BNG for post: $t(81.21) = .38$, $p = .71$, $BF_{01} = 4.13$

b. ***B"D* analysis**

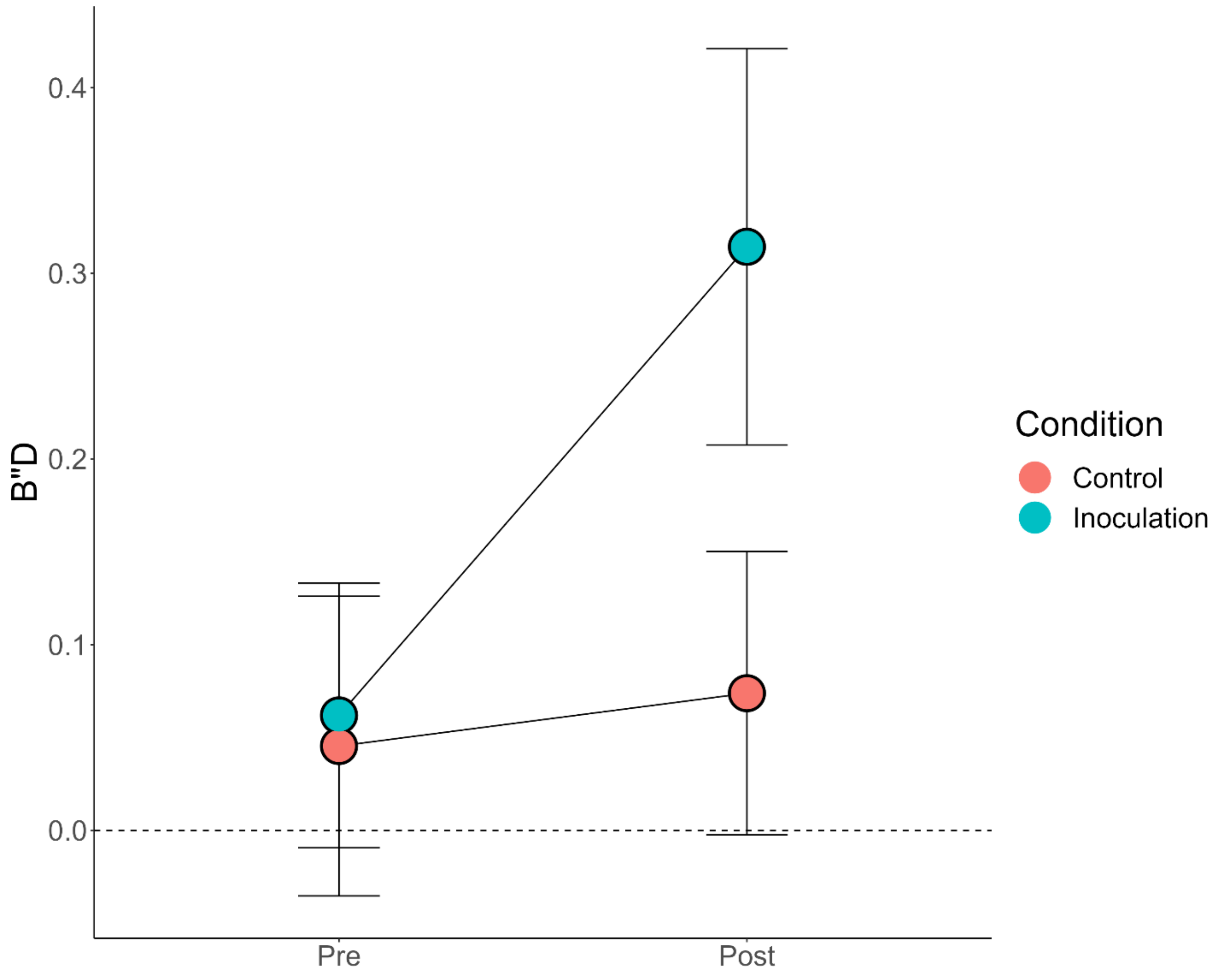

Sig. effect of condition: $F(1, 82) = 5.52$, $p = .02$

Sig. effect of pre/post: $F(1, 82) = 26$, $p < .001$

Non-sig. interaction: $F(1, 82) = 16.53$, $p < .001$

3. **Item analyses**

   a. **Individual item pre- vs. post- comparisons**

For each true/false item (109 in total), we compared the pre- and post-inoculation truth ratings for participants in the BNG condition. The objective here was to get an idea of a) variability in baseline truth ratings across items and b) potential systematic differences between true and false items that may have affected our results. The figure below shows pre- and post-inoculation ratings for true and false items, in both the BNG and control conditions:

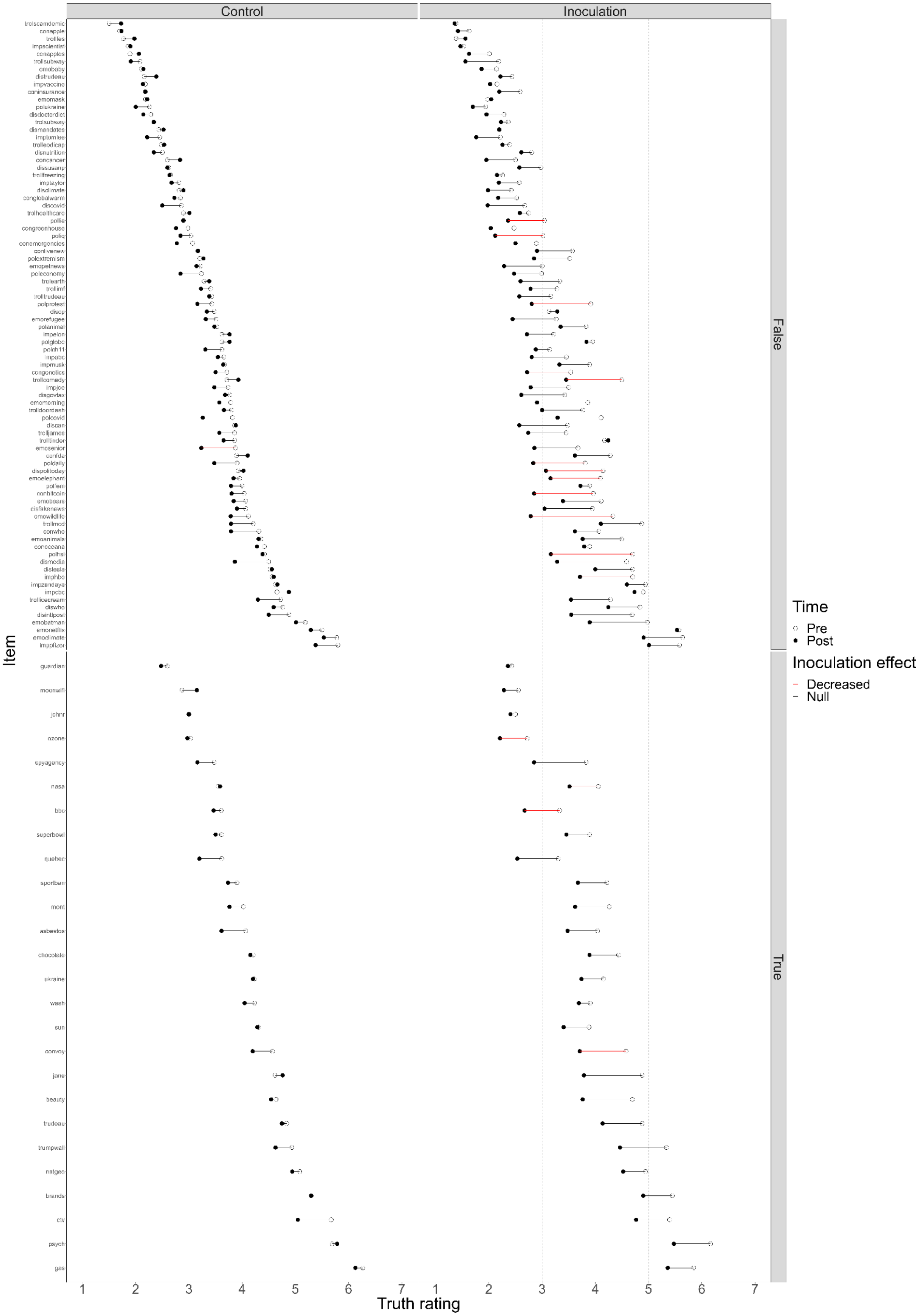
Control
Inoculation
False
True
Item
Truth rating
1
2
3
4
5
6
7
Time
Pre
Post
Inoculation effect
Decreased
Null
guardian
moonwifi
johnr
ozone
spyagency
nasa
bbc
superbowl
quebec
sportban
mont
asbestos
chocolate
ukraine
wash
sun
convoy
jane
beauty
trudeau
trumpwall
natgeo
brands
ctv
psych
gas

Note. Red lines indicate a significant decrease in truth ratings (via paired-samples *t* tests, per-comparison alpha level adjusted to .05/218 = .0002). Items ordered in descending magnitude of the inoculation truth reduction. Dashed vertical lines denote our (arbitrary) "ambiguous" region.

b. Pre-inoculation ratings

We also divided items into (arbitrary) categories based on their pre-inoculation rating, and examined the proportion of true and false items in each category:

Frequencies and Proportions			
**Pre-inoculation rating category**	**False Tweets**	**True Tweets**	**Proportion-test *p*-value**
False (≤ 3)	57/165 (0.35)	8/52 (0.15)	.006
Ambiguous (4)	100/165 (0.61)	35/52 (0.67)	.53
True (≥ 5)	8/165 (0.05)	12/52 (0.23)	.001

Note. Proportion tests compared the proportion of False and True Tweets in each ad-hoc category, against alpha = .05/3 = .017.
